# Poor Sleep Quality Increases Gestational Weight Gain Rate in Pregnant People: Findings from the MADRES Study

**DOI:** 10.21203/rs.3.rs-2944456/v1

**Published:** 2023-09-25

**Authors:** Theresa Bastain, Christine Naya, Tingyu Yang, Mario Vigil, Cindy Chen, Thomas Chavez, Claudia Toledo-Corral, Shohreh Farzan, Rima Habre, Deborah Lerner, Nathana Lurvey, Brendan Grubbs, Genevieve Dunton, Carrie Breton, Sandrah Eckel

**Affiliations:** University of Southern California; University of Southern California; University of Southern California; University of Southern California; University of Southern California; California State University, Northridge; USC Keck School of Medicine; University of Southern California; University of Southern California; University of Southern California; Keck School of Medicine, University of Southern California

## Abstract

**Background:**

Poor sleep quality is associated with weight gain in non-pregnant populations, but evidence in pregnant people is lacking. Our study examined the association between early-to-mid pregnancy sleep quality and weekly gestational weight gain (GWG) rate during mid-to-late pregnancy by pre-pregnancy body mass index (BMI).

**Method:**

Participants were 316 pregnant participants from the Maternal and Developmental Risks from Environmental and Social Stressors (MADRES) study. During early-to-mid pregnancy, participants reported their sleep quality which was used to construct four categories: very poor, poor, good, and very good. Linear growth curve models examined the association between early-to-mid pregnancy sleep quality and weekly rate of GWG (kg/week) during mid-to-late pregnancy (> 20 weeks gestation), with a three-way cross-level interaction between gestational age, sleep quality, and pre-pregnancy BMI category. Models adjusted for ethnicity by birthplace, hypertensive disorders, perceived stress score, and physical activity level.

**Results:**

Overall, poorer early-to-mid pregnancy sleep quality was associated with increased weekly weight gain during mid-to-late pregnancy. For example, amongst normal weight participants, mid-to-late pregnancy weight gain was, on average, 0.39 kg (95% CI: 0.29, 0.48) per week for those with very good sleep quality, 0.53 kg (95% CI: 0.44, 0.61) per week for those with poor sleep quality, and 0.54 kg (95% CI: 0.46, 0.62) per week for those with very poor sleep quality during early-to-mid pregnancy. This difference in GWG rate was statistically significantly comparing very good to poor sleep (0.14 kg/week, 95% CI: 0.01, 0.26) and very good to very poor sleep (0.15kg/week, 85% CI: 0.02, 0.27). This association between sleep quality and GWG rate did not statistically differ by pre-pregnancy BMI.

**Conclusion:**

Our study found very poor early-to-mid pregnancy sleep quality was associated with higher mid-to-late pregnancy GWG rate. Incorporating pregnancy-specific sleep recommendations into routine obstetric care may be a critical next step in promoting healthy GWG.

## Introduction

Gaining a healthy amount of weight during pregnancy that follows the Institute of Medicine (IOM) recommendation is important for the immediate and future health of the expectant mother and her baby.^1,2^ And yet, about one in five pregnant persons gain inadequate weight, and almost half gain excessive weight during pregnancy.^3^ This high rate of excessive gestational weight gain (GWG) is problematic, given that excessive GWG increases the risk of various pregnancy complications and long-term chronic health conditions.^4,5^

Alarmingly, rates of excessive GWG are even higher among minority pregnant persons and those with low socioeconomic status. For example, compared to the average prevalence of 35% in the United States (U.S.) population, rates are as high as 53% in low-income populations, 51% in Hispanic persons, and 61% in Black persons.^6,7^ In an attempt to curtail excessive GWG rates, current public health research and inventions have mostly focused on increasing physical activity and promoting healthy eating.^1^ Unfortunately, these efforts have not consistently led to success, especially among minority and low-income mothers.^8–10^ Thus, there is a need to identify alternative modifiable predictors of excessive GWG to improve the current prenatal care recommendations around healthy weight gain.

Poor sleep quality is a putative risk factor for weight gain and other cardiometabolic outcomes in non-pregnant populations.^11,12^ Given that almost all pregnant persons report poor sleep quality at some point during pregnancy, this may be a key health behavior in improving GWG outcomes.^13,14^ However, despite a small but growing body of research examining the effect of various indicators of sleep health on weight gain during pregnancy, there has only been three studies to date that have examined the sleep quality-GWG association.^15^ Gay et al. found poorer subjective sleep quality during the last month of pregnancy was positively associated with risk of excess GWG.^16^ A separate study by Baliero et al. showed Brazilian pregnant women with poor sleep quality during the 1st trimester gained more weight during the 2nd and 3rd trimester.^17^ Lastly, Lyu et al. reported that poor sleep quality during mid or late pregnancy were both associated with excessive GWG in Chinese women.^18^ While these three studies consistently suggest that poor sleep quality increases the risk of weight gain during pregnancy, the body of evidence is still limited in this population.

In addition to the sheer lack of research on this topic, there remains several limitations that have left sleep quality’s influence on GWG unanswered. First, most studies only assessed sleep quality during the third trimester, even though studies have found early-to-mid pregnancy sleep to be more predictive of cardiometabolic outcomes than late pregnancy sleep.^19–22^ Sleep quality assessed at the end of pregnancy can also be influenced by the amount of weight gained up to that time point, making it difficult to assess the directionality of the association between sleep and weight.^23^ Second, the majority of the studies only examine total weight gain as the outcome of interest and disregards weekly weight gain rate, even though both are important predictors of various prenatal health.^24^ And lastly, most studies did not examine pre-pregnancy BMI as an effect modifier. This leaves a significant gap in the literature, as there are some evidence that the relationship between sleep and weight gain differs by pre-pregnancy BMI,^16,25^ and the clinical implications of weight gain is meaningless without information on the patient’s pre-pregnancy BMI.

To address these gaps in the literature, our study investigated the moderated relationship between early-to-mid pregnancy sleep quality and mid-to-late pregnancy weekly GWG rate by pre-pregnancy BMI using growth curve modeling. We hypothesized that compared to pregnant participants with better sleep quality, those with worse sleep quality during early-to-mid pregnancy will exhibit higher weekly GWG rate during mid-to-late pregnancy. We also explored pre-pregnancy BMI status as a possible moderator.

## Methods

### Sample Population

The Maternal and Developmental Risks from Environmental and Social Stressors (MADRES) study is an ongoing prospective cohort study of primarily Hispanic, low-income pregnant persons and their children in Los Angeles, California. Details of the MADRES protocol have been described elsewhere.^26^ Participants were recruited from Los Angeles County + USC (LAC + USC) Medical Center, the Women’s Health Center at Eisner Health, and the South-Central Family Health Center. Inclusion criteria were: (1) < 30 weeks pregnant at the time of enrollment, (2) ≥ 18 years of age, (3) singleton pregnancy, and (4) English or Spanish speaking. Exclusion criteria were (1) HIV positive status; (2) physical, mental, or cognitive disabilities that prevent participation; (3) current incarceration; or (4) multiple gestation. Maternal consent and HIPAA authorization for abstracting electronic medical records (EMR) was obtained prior to any study assessment. The Institutional Review Board at the University of Southern California approved all aspects of this study.

For this study, we limited the analysis to participants who enrolled at less than 20 weeks gestation. The data used in this analysis consisted of 1) early-pregnancy in-person visit within 2 weeks of study recruitment, 2) mid-pregnancy telephone interview between 18–27 weeks gestation, 3) late-pregnancy visit between 30 and 34 weeks and 4) EMR abstraction from prenatal care clinic visits. All interviews were conducted in English or Spanish, and the in-person visit also included anthropometric measurements of height and weight.

## Measures

### Prenatal Sleep Quality

Overall sleep quality was assessed using the four-item Jenkin’s Sleep Questionnaire (JSQ) during early-to-mid pregnancy (mean = 15.4 weeks gestation, SD = 3.7 weeks).^27^ The JSQ is a commonly used questionnaire in epidemiologic and clinical intervention studies with pregnant populations.^28^ The JSQ consists of the following questions: “How often in the past 30 days did you have the following symptoms?”: *(1) Trouble falling asleep, (2) Waking up several times per night, (3) Trouble staying asleep and (4) Waking up after the usual amount of sleep, feeling tired and worn out*. The six response alternatives are: not at all, 1–3 nights, 4–7 nights, 8–14 nights, 15–21 nights and 22–31 nights. “Not at all” signified no sleep problems, “1–3 nights” signified rare sleep problems, “4–7 or 8–14 nights” signified occasional sleep problems, and “15–21 or 22–31 nights” signified frequent sleep problems. The response to each item is recoded on a 0- to 5-point scale and totaled to create a summary sleep score that ranges from 0 to 20 with higher scores indicating worse overall sleep quality. We then categorized the summary score into quantiles. A score of 0–3 represented very good-quality sleep, 3–6 represented good-quality sleep, 6–9 represented poor-quality sleep, and 9 + represented very poor-quality sleep.

### Maternal Height and Weight

Maternal weight and height during pregnancy were abstracted from EMRs and measured by trained staff during each of the study visits using an electronically calibrated digital scale (Tanita, Perspective Enterprises, Portage, MI) and a commercial stadiometer (Model PE-AIM-101, Perspective Enterprises) to the nearest 0.1 kg and 0.1 cm, respectively.

Self-reported pre-pregnancy weight was also ascertained through interviewer-administered questionnaires during pregnancy. If missing, then the first weight of the index pregnancy (obtained from the maternal medical records) was used in lieu of self-reported pre-pregnancy weight. Self-reported pre-pregnancy weight and height were used to calculate the pre-pregnancy BMI (kg/m^2^) and classified using CDC categories: normal weight (BMI ≥ 18.5 and < 25), overweight (BMI ≥ 25 and < 30), and obesity (BMI ≥ 30).^29^ Participants were flagged if the self-reported pre-pregnancy weight and first measured weight were more than ± 10kg discrepant, and sensitivity analyses were conducted to see if exclusion of these participants influenced study findings.

### Covariates

A list of potential covariates was identified a *priori* based on a literature review on the correlates of sleep quality and GWG. Then, bivariate analyses of the covariates with sleep quality were conducted using student’s t-tests, ANOVAs, and chi-square tests. Bivariate analyses of the covariates with GWG rate were conducted using growth curve models that included gestational age, covariate, and interaction term between gestational age and covariate. All statistical significance were examined using two-sided tests with α = 0.10. All covariates significantly associated with both sleep quality and GWG rate were included in the model.

The final list of covariates included were ethnicity by birthplace (ref: foreign-born Hispanic, US-born Hispanic, and non-Hispanic), hypertensive disorders (ref: normal, preeclampsia or gestational hypertension, and chronic hypertension), perceived stress score, and physical activity level. Perceived stress was measured using Cohen’s Perceived Stress Scale (PSS), and physical activity level was assessed via the Pregnancy Physical Activity Questionnaire (PPAQ). The PSS, PPAQ, and ethnicity by birthplace were assessed during the early-pregnancy visit. Hypertensive disorder data were collected from EMR. All continuous covariates (perceived stress and physical activity level) were centered at the mean to aid interpretation and reduce multicollinearity.^30^ While both the PSS and PPAQ were assessed during both early-pregnancy and late-pregnancy, exploratory analyses found they were similarly associated with sleep quality and GWG rate. Thus, we only used the early-pregnancy data due to higher sample size.

### Statistical Analysis

Descriptive and univariate analyses of participant characteristics were conducted to examine the distributions of variables, to understand correlation and possible multicollinearity of exposures, and to identify extreme observations. Additional analyses of residual distributions and influential points (i.e., examination of leverage, cook’s D, jackknife residuals) were performed to determine whether modeling assumptions were met before running the final model. All statistical significance were examined using two-sided tests with α = 0.05.

We examined the association between early-to-mid pregnancy sleep health and weekly GWG rate during mid-to-late pregnancy (≥ 20 weeks gestation), while simultaneously testing effect modification by pre-pregnancy BMI. We modeled this relationship using multilevel growth curve modeling. Growth curve modeling with gestational age (weeks of pregnancy centered at 20 weeks) as a predictor allows us to model the trajectory of weight gain throughout the pregnancy.^31^ Fitting the growth model within the multilevel modeling framework (level 1: weight observation, level 2: participant) takes into account the multiple repeated observations of maternal weight that are nested within each participant.^31^ It also accounts for the different number of weight observations per participant and varying time between each weight observation and handles missing values in the outcome.

We proposed a model with weight of participant *i* at each observation (*y*_ij_) as a linear function of week in pregnancy (*x*_*ij*_) with a correlated random intercept and random slope and autocorrelated residual structure to account for the nature of the dataset with repeated measures. Since we also wanted to test whether this relationship was modified by pre-pregnancy BMI, we created a three-way cross-level interaction term [sleep quality (level 2, ref: very good) × weeks in pregnancy (level 1; centered at 20 weeks) × pre-pregnancy BMI (level 2, ref: normal)].

## Results

### Sample Population

As of January 2023, of the 966 total participants, 720 participants had complete GWG data (i.e., those who had already given birth) with 10,464 individual observations of weight measurements. Of those participants, 537 were recruited before 20 weeks gestation, and 390 had completed at least one sleep health survey during early-to-mid pregnancy (< 20 weeks gestation). We then excluded any weight observations before 20 weeks gestation, as we were only interested in modeling mid-to-late pregnancy weight gain as the outcome. In addition, because we only had 8 participants who were underweight before pregnancy, we excluded them from the analysis to aid interpretation of the interaction term with pre-pregnancy BMI. We also excluded 39 participants who had preterm births. This is common practice in GWG literature, as their weight gain trajectory and behavioral risk factors are significantly different than those with term births, but we also ran sensitivity analyses by including them.^32^ Then, we excluded 19 participants who only had one weight measurement, since at least two weight measurements are necessary to examine change in weekly weight gain rate. Finally, based on exploratory data analysis, we identified and excluded 9 observations that were diagnosed as influential points as these observations were causing our model to deviate from assumptions and leading to convergence issues. Individual spaghetti plots for these 9 observations can be found in Supplemental Fig. 1 The final analytical sample consisted of 316 participants and 3,009 observations of weight measurements. The consort diagram illustrating data availability can be found in Supplemental Fig. 2. Sensitivity analyses comparing the participants in this dataset to the overall enrolled sample of 966 participants found no significant differences in participant characteristics (Supplemental Table 1).

### Descriptive Characteristics

Descriptive characteristics of the participants, overall and by pre-pregnancy BMI category, can be found in Table 1. Participants in this study were on average 28.70 years old (SD = 6.00 years), and those with overweight and obesity were older compared to those with normal pre-pregnancy BMI (F = 3.66, p < 0.05). The majority of participants were foreign-born Hispanic (44.98%) or US-born Hispanic (35.92%). A higher proportion of US-born and foreign-born Hispanic participants had overweight and obesity before pregnancy compared to those who identified as non-Hispanic ( 2 = 23.97 p < 0.01). The majority of participants (54.92%) did not finish high school, and these participants had higher rates of obesity compared to those who did finish high school ( 2 = 6.85, p = 0.03). A large majority of participants were married or living together with their partner (73.60%), but this did not differ by pre-pregnancy BMI. About one in three (33.66%) participants were nulliparous, while another third had one child and the last third already had two or more children. Multiparous participants had significantly higher rates of overweight and obesity compared to those who were nulliparous ( 2 = 20.53, p < 0.01). About one in ten (12.66%) had preeclampsia or gestational hypertension, but almost one in three (32.48%) had glucose intolerance or gestational diabetes. Both hypertensive and glucose intolerance related pregnancy complications were higher amongst those with obesity compared to those with normal BMI before pregnancy (hypertensive disorder: 2 = 13.75, p < 0.01; glucose tolerance abnormality: 2 = 22.46, p < 0.01).

Participants’ total physical activity score in early pregnancy was 302.80 MET-h/week (SD = 153.35). This value is comparable to other studies that have found average physical activity scores to range from as low as 126.0 MET-h/week (SD = 81.5)^33^ to 417.2 MET-h/week (SD = 146.2) during early-to-mid pregnancy.^34^ The average score for the PSS was 12.49 (SD = 6.38) on a range from 0 to 40; this score is comparable to other studies using the 10-item PSS in pregnant persons.^35,36^ The average score for nausea/vomiting based on the Pregnancy-Unique Quantification of Emesis and nausea (PUQE) survey was 5.85 (SD = 2.52); the PUQE score ranges from 3 (no symptoms) to 15 (maximal symptoms), and a score < 6 is considered to be mild.^37^ Lastly, about half of our participants (51.82%) were employed during early-to-mid pregnancy. None of the time-varying characteristics differed by pre-pregnancy BMI.

Overall, 23.10% of participants reported very good sleep quality (JSQ score of 0–3), 26.90% reported good sleep quality (JSQ 3–6), 22.47% reported poor sleep quality (JSQ 6–9), and 27.53% reported very poor sleep quality (JSQ > 9). While differences in sleep quality by pre-pregnancy BMI were not statistically significant, a higher proportion of those with obesity reported very poor sleep (36.04%) compared to those with overweight (19.44%) or normal BMI (26.80%).

On average, participants with normal weight before pregnancy weighed 61.5kg (SD = 0.77) at 20 weeks, and they gained 0.49 kg (SD = 0.17) per week during late pregnancy. Overweight participants weighed on average 70.9kg (SD = 0.55) at 20 weeks and gained 0.45kg (SD = 0.16) per week during late pregnancy. Participants with obesity weighed on average 87.9kg (SD = 1.53) at 20 weeks and gained 0.36kg (SD = 0.04) per week during late pregnancy.

### Bivariate Analyses

Non-Hispanic participants consistently reported poorer sleep quality ( 2 = 22.27, p < 0.01) compared to US-born and foreign-born Hispanic participants. Foreign-born Hispanic participants also had significantly lower weight at 20 weeks gestation compared to those who identified as non-Hispanic (β=−5.64, p < 0.01). Those with hypertensive disorders reported poorer sleep quality ( 2 = 12.42, p < 0.05), weighed more at 20 weeks gestation (β = 8.14, p < 0.001), and gained weight at a steeper rate (β = 0.08, p < 0.001) compared to those without hypertensive disorders. Those with very poor sleep quality had significantly higher average early-to-mid pregnancy PSS scores (F = 5.44, p < 0.01). PSS score was also positively associated with higher weekly weight gain rate during late pregnancy (β = 0.01, p < 0.01). Lastly, while early-to-mid PPAQ scores did not significantly differ by sleep quality, higher PPAQ scores were also associated with lower weekly weight gain rate (β=−0.01, p < 0.01).

### Growth Curve Linear Model

Findings from the growth curve linear model can be found in Table 2. Overall, we found those with poorer sleep quality during early-to-mid pregnancy exhibited higher weekly weight gain rate during mid-to-late pregnancy. In the reference group (participants with normal pre-pregnancy BMI), we found those who reported poor early-to-mid pregnancy sleep quality gained on average 0.14 kg (95% CI: 0.01, 0.26) more during mid-to-late pregnancy compared to those who reported very good sleep quality. Those who reported very poor early-to-mid pregnancy sleep quality gained on average 0.15kg (95% CI: 0.02, 0.27) more during mid-to-late pregnancy compared to those who reported very good sleep quality. We did not find significant difference in weight gain rate when comparing those with very good sleep quality to good sleep quality (GA × Good sleep quality: 0.12 [95% CI: 0.00, 0.25]).

Furthermore, the relationship between sleep and weekly weight gain did not significantly differ by pre-pregnancy BMI, as can be seen from the null three-way interaction terms. For example, amongst those with normal pre-pregnancy BMI, those who reported very poor sleep quality gained on average 0.15kg (95% CI: 0.02, 0.27) more than those with very good sleep quality. Amongst those with overweight pre-pregnancy BMI, those who reported very poor sleep quality gained on average 0.10kg (95% CI: 0.07, 0.26) more than those with very good sleep quality. The interaction term representing the difference between these two slopes (GA × Very poor sleep quality × Overweight BMI: −0.05 [95% CI: −0.22, 0.12]) is null.

**Figure 1.**
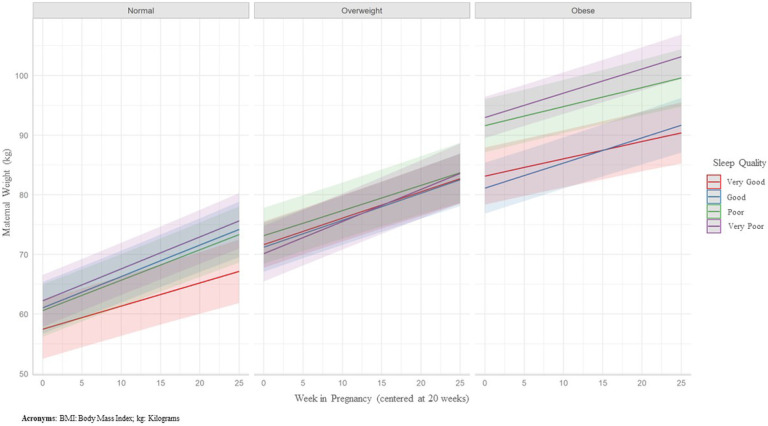
Early-to-Mid Pregnancy Sleep Quality and Mid-to-Late Pregnancy Weight Gain by Pre-Pregnancy BMI Status

To put this into context, the IOM recommends that pregnant persons with normal pre-pregnancy BMI gain 0.42 (0.35–0.50) kg per week, overweight pre-pregnancy BMI gain 0.28 (0.23–0.33) kg per week, and obesity pre-pregnancy BMI gain 0.22 (0.17–0.27) kg per week during the 2nd and 3rd trimester. Participants with normal pre-pregnancy BMI with very good quality sleep fell into this range, gaining on average 0.39 kg (95% CI: 0.29, 0.48) per week. However, all other participants gained above this recommended amount with those with very poor sleep quality gaining about 0.12kg − 0.26kg above the recommended rate, depending on pre-pregnancy BMI. The average rates of mid-to-late pregnancy weekly weight gain by early-to-mid pregnancy sleep quality can be found in Table 3 and Fig. 1.

Sensitivity analyses including the participants with preterm births did not significantly change results; given that their weight gain trajectory and behavioral risk factors are significantly different than those with term births we implemented what is more common in the GWG literature and excluded these participants. Excluding participants with ± 10kg discrepancies between self-reported pre-pregnancy weight and first measured weight did not change the results, and thus we did not eliminate these participants from the analysis. Lastly, there was one participant (four weight observations) who had a clinical diagnosis of sleep disturbances but excluding this participant did not change the results.

## Discussion

This study investigated the effect of early-to-mid pregnancy sleep quality on GWG rate during mid-to-late pregnancy. We found pregnant persons who reported poorer quality sleep gained more weight on a weekly basis. This association did not differ across pre-pregnancy BMI status. The results of our analysis agree with a large body of evidence in non-pregnant populations that have found poor sleep quality to predict increased weight gain.^38,39^ Our findings are somewhat in line with the previous three studies that found poorer subjective sleep quality during pregnancy increased weight gain.^16–18^ However, these studies only examined total weight gain as the outcome, as opposed to weight gain rate, and assessed sleep quality at different points during pregnancy compared to our study. This discrepancy makes it dificult to directly compare these findings to ours.

While the current body of literature supporting the association between sleep quality and increased GWG is small, there are well-established psychological, behavioral, and biological mechanisms that explain this relationship. Pregnant persons often cite sleep difficulties as a source of stress,^40–42^ and while the findings are somewhat mixed, various studies have linked stress exposure to risk of excessive GWG or increased total weight gain in general.^43,44^ In both non-pregnant and pregnant populations, sleep and metabolism are intimately linked through the hypothalamic-pituitary-adrenal (HPA) axis activation.^45–47^ Poor sleep is associated with maladaptive changes to the HPA axis, which in turn leads to neuroendocrine dysregulation that are related to fat accumulation, inflammation, insulin sensitivity, and energy metabolism.^48^ Poor sleep can also alter food intake through hormonal (e.g., increased ghrelin and decreased leptin levels increases appetite)^11,15,39^ and psychological pathways (e.g., people are more likely to consume “comfort foods” as a coping mechanism).^49,50^ Furthermore, a recent systematic review found significant evidence showing that poor prenatal sleep quality was associated with lower levels/duration of physical activity.^15^ Taken together, a pregnant person with poor sleep quality may experience physiological changes and negative affect in ways that perpetuate obesogenic behaviors.

## Limitations

One of our study limitations is our lack of objective (i.e., device-derived) sleep data. In the sleep literature, it is usually recommended to include both objective and subjective measures of sleep in the same study.^51^ It’s widely known that subjective sleep (e.g., self-reported measurements of sleep duration, quality, disruption via questionnaires) and objective sleep (e.g., polysomnography, actigraphy, bed sensors) often do not agree with one another, but both types of sleep measures are independently associated with health factors.^52–54^ Given that the correlation between subjective and objective measures of sleep varies by perinatal mood disorders, we cannot conclude how the lack of objective sleep data may have biased our results.^54,55^ Furthermore, pre-pregnancy BMI is based on self-reported weight, and therefore, may be at risk of recall bias, even with the extra precaution taken to conduct sensitivity analyses with any participants with a +/− 10kg discrepancy between the self-reported pre-pregnancy weight and first measured prenatal weight.

## Conclusion

Our findings identify sleep health during pregnancy as a promising intervention target to curtail excessive GWG rates. In non-pregnant populations, improving sleep quality as part of weight management interventions have led to success amongst those with obesity or overweight BMI.^56^ Sleep is also proven to be extremely amenable via interventions such as sleep hygiene education, mindfulness-based practices, and cognitive behavioral therapy, even during the perinatal period.^57^ Furthermore, unlike current GWG interventions aimed at solely increasing physical activity or improving nutrition, sleep interventions could be more widely accepted amongst persons of color. Qualitative studies have found Black, Hispanic, and Asian cultural beliefs view sleep and rest as some of the most important behaviors for pregnancy health, while many believe physical activity or decreased caloric intake can adversely affect the health of the baby.^58,59^ And yet, current guidelines for perinatal care from the American College of Obstetricians and Gynecologists includes counseling on physical activity and nutrition but not sleep hygiene.^60^ The development of pregnancy-specific sleep recommendations to be incorporated into routine obstetric care is a critical next step in promoting healthy GWG.

## Data Availability

The datasets generated during and/or analyzed during the current study are not publicly available due to personally identifiable information but are available from the corresponding author on reasonable request.
